# The Nutraceutical Value of Olive Oil and Its Bioactive Constituents on the Cardiovascular System. Focusing on Main Strategies to Slow Down Its Quality Decay during Production and Storage

**DOI:** 10.3390/nu11091962

**Published:** 2019-08-21

**Authors:** Lorenzo Flori, Sandra Donnini, Vincenzo Calderone, Angela Zinnai, Isabella Taglieri, Francesca Venturi, Lara Testai

**Affiliations:** 1 Department of Pharmacy, University of Pisa, via Bonanno 6, 56126 Pisa, Italy; 2 Department of Life Sciences, University of Siena, Via A. Moro 2, 53100 Siena, Italy; 3 Interdepartmental Research Centre, Nutraceuticals and Food for Health, University of Pisa, Via del Borghetto 80, 56124 Pisa, Italy; 4 Department of Agriculture, Food and Environment, University of Pisa, Via del Borghetto 80, 56124 Pisa, Italy

**Keywords:** olive oil, polyphenols, vitamin E, oleic acid, shelf life, nutraceutical value, storage temperature, packaging, light exposure

## Abstract

Cardiovascular diseases represent the principal cause of morbidity and mortality worldwide. It is well-known that oxidative stress and inflammatory processes are strongly implicated in their pathogenesis; therefore, anti-oxidant and anti-inflammatory agents can represent effective tools. In recent years a large number of scientific reports have pointed out the nutraceutical and nutritional value of extra virgin olive oils (EVOO), strongholds of the Mediterranean diet, endowed with a high nutritional quality and defined as functional foods. In regard to EVOO, it is a food composed of a major saponifiable fraction, represented by oleic acid, and a minor unsaponifiable fraction, including a high number of vitamins, polyphenols, and squalene. Several reports suggest that the beneficial effects of EVOO are linked to the minor components, but recently, further studies have shed light on the health effects of the fatty fraction and the other constituents of the unsaponifiable fraction. In the first part of this review, an analysis of the clinical and preclinical evidence of the cardiovascular beneficial effects of each constituent is carried out. The second part of this review is dedicated to the main operating conditions during production and/or storage that can directly influence the shelf life of olive oil in terms of both nutraceutical properties and sensory quality.

## 1. Introduction

Cardiovascular diseases (CVDs) are a major health problem and, to date, the principal cause of morbidity and mortality worldwide [1]. The main condition that exposes people to CVD is represented by atherosclerosis, defined as a progressive inflammatory process caused by an excessive cholesterol deposition in the arterial walls. It is well-known that oxidative stress is strongly implicated in the pathogenesis of atherosclerosis, and oxidized low density lipoproteins (ox-LDL) play a critical role [2,3,4]. Indeed, reactive oxygen species (ROS) can rapidly inactivate nitric oxide (NO) and form reactive nitrogen species (RNS) that damage vascular endothelial cells, creating a prothrombotic environment and an associated inflammatory condition. Therefore, anti-oxidant and anti-inflammatory agents can represent effective tools against atherosclerosis and, consequently, CVD [5].

Indeed, in heart failure, inflammatory processes associated with fibrosis and alteration of angiogenesis lead to cardiac hypertrophy [6]. Moreover, several studies have shown that cardiac dysfunctions such as myocardial infarction are associated with an increase of myocardial oxidative stress [7]. Finally, coronary heart diseases can be deeply influenced by diet habits, particularly the intake of saturated fatty acids [8].

In this context, lifestyle and dietary modifications are strongly recommended as an efficient, early interventional approach to changing these modifiable risk factors, acting especially on ROS and inflammatory markers.

In recent years a large number of scientific reports have pointed out the nutraceutical and nutritional value of the Mediterranean diet, suggesting that its consumption contributes to the reduction in the incidence of oxidative- and inflammatory-related pathologies, such as cardiovascular diseases and cancer. Virgin and extra virgin olive oils (EVOO) are a stronghold of the Mediterranean diet and have been described as functional foods endowed with a high nutritional quality [9,10,11,12,13]. Indeed, the bio-functional components of EVOO show positive effects on genes involved in the pathogenesis of most prevalent age- and lifestyle-related human conditions, pointing to a role for these molecules as natural homeostatic and even hormetic factors in applications such as prevention agents used to treat conditions of premature and pathologic aging [14].

**Figure 1:**
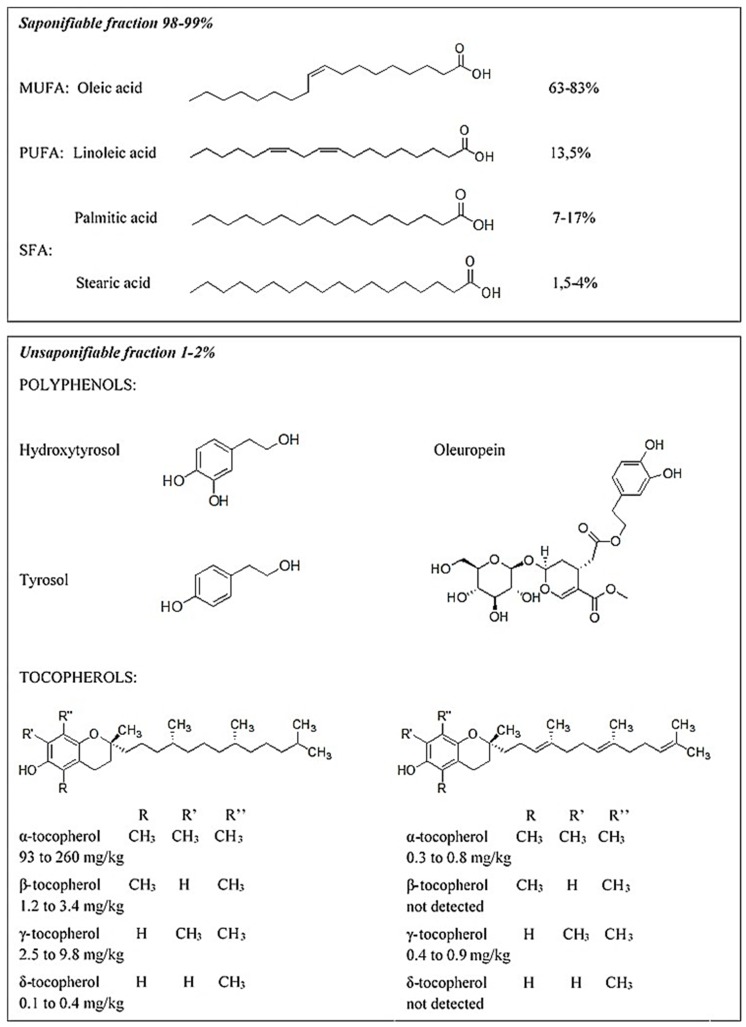
Chemical structure and relative amounts of the main constituents of EVOO.

Olive oil (OO) is a food composed of a major saponifiable fraction (about 98–99%) represented by oleic acid (55–83%) and other saturated and unsaturated acids (linoleic, palmitic and stearic acids, 3–21%), and of a minor unsaponifiable fraction (about 1–2%), including a high number of vitamins (α-, β-, γ- and δ- tocopherols), polyphenols (mainly tyrosol, hydroxytyrosol, and oleuropein) and squalene [15,16] (Figure 1).

Several reports suggest that the beneficial effects of EVOO are linked to the minor components and in particular to polyphenols; however, further studies have recently shed light on the health effects of the fatty fraction and the other constituents of the unsaponifiable fraction. 

Indeed, the concept that saturated fatty acids (SFA) increase serum cholesterol and induce inflammation and insulin resistance, thus contributing to the risk of atherosclerosis and CVD, is generally accepted; on the other hand, various translational studies identify a protective role for unsaturated oils, monounsaturated fatty acids (MUFA), and more widely for polyunsaturated fatty acids (PUFA). 

Considered as a whole, this evidence shows that EVOO is a functional food endowed with a healthy profile and the widely-studied phenolic component, as well as tocopherols and the MUFA (represented by oleic acid) fraction, can contribute in different ways and act on different types of molecular targets to ensure interesting pleiotropic effects.

In this regard, in 2004, based on numerous clinical trials carried out in the past few decades [17,18,19,20,21], the US Food and Drug Administration (FDA), and more recently the European Food Safety Authority (EFSA), authorized the health claims for olive oil, suggesting a dose of 20–23 g/day as a replacement for the same amount of saturated fats to reduce the risk of coronary diseases [22,23]. 

However, the quality of EVOO depends on a process that begins with the olive ripening and finishes with the packaging. Thus, agronomical practices, raw materials, harvesting, fruit storage, and extraction technology, and also oxygen, light, and temperature during storage, have to be considered in order to correctly estimate the nutraceutical, nutritional, and sensorial value.

In this context, the aim of this review has been twofold: firstly, an extensive analysis of clinical and preclinical evidence of cardiovascular beneficial effects of both unsaponifiable and saponifiable fractions of EVOO has been carried out; in the second part of the paper, the main operating conditions adopted during EVOO production and/or storage have been pointed out and critically discussed in order to highlight their influence on the concentration of health compounds in extracted oil as well as on their preservation during oil storage. 

## 2. Methodology

A search was conducted from January 2010 to June 2019 using the search terms listed in Table 1, mainly in the following bibliographic databases: PubMed, Science Direct, and Web of Science. The searched keywords were not established in advance but emerged gradually during the extensive reading process that preceded the drafting of this review:

Starting from the reference list of the manuscripts selected in the predetermined timespan (January 2010–June 2019), we also included papers published before this period if they were useful to better describe our topic.

## 3. Unsaponifiable Fraction

### 3.1. Polyphenolic Components

Secoiridoid derivatives such as oleuropein (Ole), hydroxytyrosol (3,4-dihydroxyphenylethanol, HT), and tyrosol ((2-(4-hydroxyphenyl)-ethanol, Tyr) are the major OO phenolic compounds (Figure 1). OO polyphenols exert a wide range of biological effects, including cardio-protective, neuro-protective, anticancer, antimicrobial, and anti-inflammatory effects [24,25,26]. At the molecular level, their biological activities are associated with either anti-oxidant activity, or with the regulation of a variety of signaling molecules involved in inflammation, cell adhesion, cell growth, apoptosis, and aging [27,28,29,30].

Ole is the OO polyphenol with a catechol functionality (1,2-dihydroxybenzene moiety) associated with its health-protective effects [31]. After being adsorbed, the Ole-aglycone (derived by gastric hydrolysis of Ole and by the native Ole-aglycone present in OO) is hydrolyzed into HT and elenolic acid, and further metabolized [32]. In the intestine, the microflora decomposes Ole into HT, and it is the latter that has the main biological effect on the cells of the large intestine [32,33].

HT is the major bioactive compound in OO. It is a phenolic alcohol with a poor bioavailability (plasma half-life of 1–2 min) due to its low hydrophilic solubility and its extensive first-pass phase-I and phase-II metabolism in the gut and liver [34]. It is worth noting that HT derivatives of phase-II metabolism, with methyl/sulphate/glucuronide functional groups, did not seem to inhibit the biological activity of the HT [35]. After being adsorbed, HT and its derivatives are quickly incorporated in plasmatic High-Density Lipoproteins (HDLs) and acts as a cardiovascular protector [36,37]. 

Tyr is a cellular stable antioxidant agent that accumulates in cell cytoplasm. It is extensively metabolized, and its bioavailability is poor compared to that of its derivatives [35,38]. Similar to Ole, the absorbed Tyr could be converted into HT in the liver by phase-I metabolism or in the intestine by gut microbiota [34,39,40]. The most abundant metabolites of Tyr, 4’-O-glucuronide and 4’-O-sulphate, are derived from phase-II metabolism.

#### 3.1.1. Beneficial Effects of Polyphenols: Clinical Evidence

The cardioprotective effects of OO polyphenols have been investigated in numerous clinical studies (Table 2). The results of the clinical trial “European Study of the Antioxidant Effects of Olive Oil and its Phenolic Compounds on Lipid Oxidation” (EUROLIVE) has been a key report in the research of virgin OO polyphenols on human health, prompting the EFSA to publish the health claim on the cardioprotective role of HT [41,42]. More recent clinical trials have supported these results, observing that the consumption of HT-enriched biscuits or virgin OO enriched with HT and derivatives reduce plasma ox-LDL [43,44,45,46]. It is also noteworthy that EFSA’s claim only focuses on the capability of HT to protect LDL from oxidation, the clinical relevance of which is still unclear. 

Other trials with OO or olive extracts enriched with Ole and/or HT confirmed their cardio-protective contribution [47,48,49,50]. The European Prospective Investigation into Cancer and Nutrition (EPIC) and the Prevención con Dieta Mediterránea (Prevencion con Dieta Mediterranea, PREDIMED) trials showed that the daily consumption of OO significantly decreases the incidence of several chronic diseases such as cardiovascular, metabolic, immune-inflammatory disorders, and cancer [51,52,53,54,55]. However, as virgin OO contains other phenolics and bioactive compounds, the protective effects reported in these studies cannot be exclusively attributed to HT and its derivatives or precursors [56]. 

The consumption of HT per se has been investigated in several clinical randomized trials with discordant results [44,57,58,59] (see also Table 2). A Phase 3 interventional study on the efficacy and safety of HT and Vitamin E in children with non-alcoholic steatohepatitis is currently underway (Trials.gov Identifier: NCT02842567), in addition to a Phase 2 and 3 trial on the efficacy of HT (25 mg orally, once daily for 1 year) on mammographic density in women at high risk of developing breast cancer (ClinicalTrials.gov Identifier: NCT02068092).

#### 3.1.2. Beneficial Effects of Polyphenols: Preclinical Evidence

Many in vivo studies on animal models of atherosclerosis confirmed the beneficial effect of OO polyphenols on the cardiovascular system. In Wistar rats, olive leaf extract rich in Ole, Ole-aglycone, and HT lowered serum cholesterol, triglycerides, and LDL levels, and increased HDL levels, decreased the lipid peroxidation process, and enhanced antioxidant enzyme activity [76]. Furthermore, in ApoE−/− mice, 10 mg/kg/day of HT derivatives for 12 weeks downregulated the expression of vascular cell adhesion molecules involved in early atherogenesis, such as E-selectin, VCAM-1, MCP-1, ICAM-1, and F4/80 macrophage marker expression compared with the control group [77]. OO polyphenols also exerted protective effects on the progression of non-alcoholic fatty liver disease (NAFLD) to fibrosis in a mouse model [78,79,80], and exerted anti-obesity effects by regulating the expression of genes involved in adipogenesis in the visceral adipose tissue of high-fat diet-fed mice [81,82]. In particular, HT supplementation prevented early inflammatory processes causally associated with the onset of insulin resistance and steatosis [81], activated transcription factors such as PPAR-α, -γ and Nrf2, and inhibited NF-κB and SREBP-1c as well as their target genes [83,84,85,86]. Furthermore, olive leaf extract containing Ole and HT reversed the chronic inflammation and oxidative stress, and normalized cardiovascular, hepatic, and metabolic signs in Wistar rats with signs of metabolic syndrome [87]. 

Besides the above, in in vitro studies, Ole and HT have been shown to exert several protective effects on a model of atherosclerosis inhibiting endothelial activation and monocyte-endothelial cell adhesion [88]. HT has been shown to enhance the expression of genes involved in cholesterol efflux and, in endothelial cells (EC) exposed to inflammatory stimuli or ROS, that of antioxidant enzymes [89]. Indeed, the pre-treatment of endothelial cells with HT suppressed inflammatory angiogenesis, reduced mitochondrial superoxide production and lipid peroxidation, and increased Superoxide Dismutase (SOD) activity [90]. Similarly, the glucuronide forms of HT showed antioxidant activity in the HepG2 cell line [91], in red blood cells, and in kidney epithelial cells [56,92]. Moreover, HT and Tyr sulphates have recently been shown to protect Caco-2 cells from oxidative damage by ox-LDL if compared with the parent compounds [38,56,93]. The sulphate metabolite of HT, HT-3Os, also inhibited the mesenchymal phenotype of ECs exposed to IL-1β, and restored the EC phenotype [30]. Consistently, in another study, a mixture of HT metabolites with 80% HT-3Os showed a significant decrease of inflammation biomarkers in ECs, leading to an improvement of endothelial dysfunction [94]. Like HT, Tyr also reduced oxidative modifications to HDL, thus promoting cholesterol efflux [95]. It also inhibited leukotriene B4 production, exerting a protective role on EC function [96], and protected the heart and brain from ischemia related stress [97,98]. OO polyphenols also showed protective effects on in vitro models of obesity. Indeed, HT inhibited lipogenesis [99] and regulated genes related to adipocyte maturation and differentiation [100,101]. Similarly, Tyr downregulated lipid synthesis in primary-cultured rat-hepatocytes [102] and also exerted beneficial effects in NAFLD, increasing hepatic cystathionine β-synthase and cystathionine γ-lyase expression and hydrogen sulphide synthesis in high-fat diet-fed mice [103]. Furthermore, HT acted as a caloric restriction mimicker in muscle, brain, fatty tissue, and the kidney through the production and activation of sirtuins [25].

### 3.2. Vitamin E

Vitamin E consists of a family of eight different compounds: four tocopherols (α-, β-, γ-, and δ-tocopherol) and four tocotrienols (α-, β-, γ-, and δ-tocotrienol) [104]. These molecules have a common structure composed of a head known as a chromanol ring and tail called phytyl tail. The chromanol ring has one hydroxyl group and two methyl groups, the position of which is different in each type of tocopherol. The difference between tocopherols and tocotrienols lies in the tail region, as the latter have three double bonds in their phytyl tails [105] (Figure 1). 

Tocopherols are absorbed along with dietary fats in the intestine and are secreted as chilomicron particles that are transported into the adipose tissue, skin, muscles, bone marrow, and brain. α-Tocopherol is preferentially bound to α-tocopherol transfer protein, which protects it from catabolic enzymes in the liver. Other tocopherols, especially γ-, β-, and δ-tocopherol, undergo ω-hydroxylation, oxidation, and β-oxidation in the liver to generate 13’-hydroxychromanols and carboxychromanols, which have potent antioxidant properties and a strong radical-scavenging action. The oxidative action of the radical-scavenger species of tocopherols is caused by the donation of the hydrogen ion from the phenol group on the chromanol ring. These metabolites have been shown to inhibit the cyclooxygenase (COX)-2 and 5-lipoxygenase (LOX) pathways more strongly than the non-metabolized forms. This could be the reason for a stronger anti-inflammatory and antioxidant action than γ-tocopherol compared to α-tocopherol. γ-Tocopherol has a unique non-substituted C-5 position for trapping electrophiles, including the RNS [105].

#### 3.2.1. Beneficial Effects of Vitamin E: Clinical Evidence 

An inverse association has been suggested between the intake of vitamin E from food and/or supplements and the risk of CVD. Several cohort studies reported promising and significant results about reduction of the ischemic cardiomyopathy risk [7,106,107,108,109,110], as well as coronary artery disease [108] myocardial infarction [111] and mortality due to heart failure [112] in subjects taking vitamin E supplements. In another study, people taking vitamin E for more than 4 years showed a 59% reduction in mortality for coronary heart disease [108]. Moreover, the Cambridge Heart Antioxidant Study showed that treatment with α-tocopherol (400–800 mg/dL) reduced the risk of myocardial infarction in patients with coronary atherosclerosis [111]. Interestingly, a study of secondary prevention with antioxidants demonstrated that the administration of α-tocopherol (800 mg/dL) significantly reduced the endpoint of myocardial infarction (fatal and non-fatal) and stroke, in patients suffering from renal disease in the final-stage [113]. Several clinical investigations have also focused on the effect of γ-tocopherol, which is inversely correlated with coronary artery disease [114,115] alone or mixed with other analogue condition. Studies using supplementation of γ-tocopherol alone and in combination with α-tocopherol revealed a reduction in the biomarkers of oxidative stress in patients with metabolic syndrome [116]. In contrast, the effect of tocotrienols in a randomized controlled trial showed no significant change either in vascular function or in CVD risk factors [117]. 

Despite promising results against cardiovascular complications, some clinical studies have reported controversial data [118,119]. It is worth noting that no significant correlation between vitamin supplementation E and the incidence of ischemic CVD was confirmed in the Supplementation en Vitamines et Mineraux Antioxydants Study. Similarly, the collaborative Japanese cohort study found no significant association between vitamin A and E intake and stroke, or coronary heart disease and CVD mortality [120]. Finally, another research group studied the effects of α-tocopherol and the combination of PUFA in patients with myocardial infarction. Despite the beneficial effects of dietary supplementation with PUFA against cardiovascular events, the vitamin E group showed no improvement [121]. Moreover, a study on the evaluation of cardiac prevention showed that 400 IU of α-tocopherol administered daily for 4–6 years had no beneficial effect on cardiovascular outcomes in a population of high-risk elderly patients [122,123]. Another publication reported no significant correlation between vitamin E and mortality in patients with a high cardiovascular risk [124]. 

Table 3 contains a summary of the main clinical studies in which the effects of vitamin E have been evaluated.

#### 3.2.2. Beneficial Effects of Vitamin E: Preclinical Evidence

In regard to preclinical evidence, α-tocopherol decreases lipid peroxidation and platelet aggregation [126]. Furthermore, the adhesion of monocytes to endothelial cells in vitro decreases, possibly through the inhibition of NFkB [127]. α-Tocopherol inhibits monocyte-mediated production of superoxide and platelet aggregation and their adhesion. α-Tocopherol also has an interesting regulating action on vascular homeostasis by increasing Nitric Oxide (NO) production and preserving endothelium-dependent vasodilatation [128,129]. All these properties are also shown by γ-tocopherol.

Studies on cell cultures and animals have confirmed the preventative role played by α-tocopherol in CVD because of its important effects in modulating specific signaling pathways and gene expression. A recent paper demonstrated that α-tocopherol was able to inhibit Protein Kinase C (PKC), followed by a reduction in the proliferation of smooth muscle cells both in rat aorta and in humans [130,131,132]. α-Tocopherol is an effective inhibitor of superoxide production in human adherent monocytes, compromising the assembly of Nicotinamide Adenine Dinucleotide Phosphate (NADPH)-oxidase and attenuating p47 membrane translocation and its phosphorylation [133]. Other results showed that the treatment of macrophages and monocytes with α-tocopherol inhibited the absorption of ox-LDL by reducing the expression of CD36 [134,135]. Subsequently it has been reported that α-tocopherol reduced the formation of foam cells, thus preventing the induction of NFkB and the expression of P-selectin in macrophage cell lines [136]. The atheroprotective effects have also been tested on animal models using diets based on olive oil, palm oil, and sunflower oil, observing a reduced extension of the atherosclerotic lesion in the aorta of treated mice [137]. Moreover, these animals showed an attenuation of the progression of the lesions in the ascending aorta, the aortic arch, and the descending aorta [138]. Other research groups have reported that vitamin E supplementation was effective in reducing atherosclerotic lesions in Knock-Out (KO) mice for LDL receptors (LDLR -/-) [139]. The effect of vitamin E was also observed in the reduction of the fibrotic area of the aorta demonstrated by measuring the collagen accumulation and dissociation of elastic fibers in an in vivo model of atherosclerosis induced by homocysteine and cholesterol [140]. In vivo studies showed that α-tocopherol supplementation reduced the expression of CD36, which is recognized as the most important CVD-related scavenger receptor and plays an essential role in the atherogenic process (in particular, it is closely related to cell formation foam) and is localized in monocytes, macrophages, endothelia, and smooth muscle cells [141]. It has also been shown that α-tocopherol is able to prevent the formation and extension of cholesterol-induced atherosclerotic lesions by decreasing the activity of PKC in models of rabbits fed with a cholesterol-rich diet [142]. Vitamin E also reduced the development of atherosclerosis through the induction of PPARγ and Nrf2 followed by the enhancement of their downstream targets [143].

The anti-inflammatory effects of α-tocopherol have been also reported in cellular and animal models. An important part of its anti-inflammatory role occurs through the inhibition of NFkB and the reduction of PKC activity and of the biosynthesis of adhesion molecules [144,145]. A modulatory effect by α-tocopherol during inflammatory processes was identified in the decrease of cytokines (IL-1β, IL-6, IL-8) and tumor necrosis factor α (TNF-α) release and inhibiting the 5-LOX pathway [146].

Furthermore, it has been hypothesized that early vitamin E (25 mg/kg/day) supplementation reduced mortality following acute myocardial infarction induced by occlusion of the left anterior descending coronary artery in male Wistar rats [6,147].

Moreover, other experimental investigations have defined a beneficial role of vitamin E by reducing the apoptotic activity of cardiomyocytes [148]. Indeed, a diet enriched with vitamin E showed a cardioprotective effect in a condition of streptozotocin-induced diabetic heart failure in rats [149]. Other studies have shown that α-tocopherol supplementation prevented the cholesterol-mediated damage of cardiomyocytes by reducing the expression of LXRα and increasing the levels of ABCA1 in hypercholesterolemic rabbit models [150].

## 4. Saponifiable Fraction

### 4.1. MUFA

It is well-known that SFAs are implicated in cardiovascular morbidity and mortality. Indeed, an increase thereof is associated with the pathogenesis of obesity and of obesity-related diseases [151,152]. Moreover, it has been found that there is a positive correlation between SFAs and the severity of hypoxic-damage in the brain, and finally, a direct proportionality emerged between the intake of SFAs and markers of acute myocardial infarct [153,154,155]. 

Instead, with regard to PUFAs, it is a well-established fact that they have a positive impact on lipid profile and on systemic inflammatory markers [156], especially with regards to omega 3 [157]; nevertheless, only little and often unclear evidence has been published on the beneficial effects of MUFA and particularly on the most widely represented MUFA in olive oil—oleic acid (Figure 1).

In humans, oleic acid is naturally present as an ester and is mainly found in adipose tissue [158]. In the diet, oleic acid is the most important MUFA. Indeed it is the main component of the saponifiable fraction of olive oil, and on this basis, it is a fundamental component of the Mediterranean diet. However, other kinds of vegetables may represent an effective source of it; worthy of mention is oil of canola and flaxseed, which contain high amounts of oleic acid, similar to that of olive oil [159]. 

Usually, the total intake of oleic acid in adults varies between 12% and 18% of energy, but it is higher in Southern European countries (up to 29%) like Greece, Italy or Spain that are traditionally large consumers of olive oil [21]. 

#### 4.1.1. Beneficial Effects of Oleic Acid: Clinical Evidence

Interestingly, several years ago, Lopez-Huertas carried out an examination on scientific evidence regarding the effects of milk enriched with PUFA (in particular, omega 3) and/or oleic acid. In particular, the authors selected nine controlled intervention studies on enriched milk in which healthy volunteers, subjects with increased risk factors, and patients with CVD were enrolled. Overall, the main effects observed were reductions in blood lipids, mainly cholesterol, LDL, and triglycerides. Nevertheless, it should be noted that in all studies, oleic acid was used alone. Indeed, it was always associated with omega 3, so any beneficial effects on lipid profile were certainly due, at least in part, to their presence [160].

It is worth noting that the multicenter study PREDIMED, carried out in Spain, demonstrated, after 4.8 years of observation, a lower cardiovascular risk and a reduced incidence of major cardiovascular events in the group assigned to the Mediterranean diet plus EVOO or nuts [161].

Very recently, a randomized crossover trial (NCT02145936) has been carried out to compare several types of SFAs, varying in chain length (in particular palmitic acid and stearic acid), with MUFA (i.e., oleic acid) on cardiometabolic risk factors. In particular, for a period of five weeks, postmenopausal women with mildly hypercholesterolemia were given a diet enriched in SFAs or MUFA. Any type of diet had significant effects on systemic and vascular inflammatory markers, coagulation markers, T lymphocytes proliferation, or glucose homeostasis. The main finding of the trial was that oleic acid enriched diets produced a lower fecal total secondary bile acid (SBA) concentration than palmitic acid, hypothesizing that its hypocholesterolemic effects may be mediated through differential effects on the bile acid metabolism; indeed, SBA concentrations are assessed as a potential mechanism for plasma cholesterol responses [162].

Conversely, a previous prospective longitudinal cohort study showed that oleic acid, like SFAs, was linked to left ventricular hypertrophy, a main cause of cardiovascular death [163]. 

Table 4 summarizes the main clinical studies in which the beneficial effects of oleic acid have been evaluated.

#### 4.1.2. Preclinical Evidence of Beneficial Effects of Oleic Acid

Beside these, Perdomo et al., in 2015, also demonstrated that oleic acid played protective effects against insulin resistance by improving endothelial dysfunction in response to pro-inflammatory stimuli. In fact, cardiomyocytes exposed to insulin treatment significantly increased Akt phosphorylation and then inactivated AMP-Activated Protein Kinase (AMPK) through self-dephosphorylation. On the other hand, the exposition of vascular or endothelial cells or cardiomyocytes to oleic acid before treating with palmitate or TNF α prevented insulin resistance through the modulation of pathway downstream to NFkB. Moreover, the authors demonstrated for the first time that oleic acid significantly reduced the expression of adhesion molecules (ICAM-1 and MCP-1) induced by inflammatory stimuli on endothelial cells. On the other hand, in vascular cells, oleic acid prevented proliferation and apoptosis, suggesting that it could improve the growth and stability of atherosclerotic plaque, thus preventing underlying complications such as thrombosis [165].

Opposite results come from the study by Chan, who observed that oleic acid, in vascular aortic smooth muscle cells, promoted the enhancement of matrix metalloproteinases (MMPs) through SIRT1 downregulation. In particular, MMP-1 and MMP-3 are responsible for collagen and elastin digestion, thereby rupturing atherosclerotic plaques. SIRT1 plays a critical role in the modulation of MMPs under oleic acid-stimulus; indeed, it was assumed that oleic acid inhibited the SIRT1 enzyme and thus promoted NFkB activation. Besides this, an iNOS-mediated NO production has been also observed, leading to speculation that oleic acid, at the atherosclerotic plaque level, inhibited the SIRT1 axis, which involves the activation of NFkB expression and iNOS activity, which in turn influences the production of MMPs [166].

Conversely, Lim and colleagues demonstrated that oleic acid was able to directly activate the SIRT1 enzyme, thus modulating AMPK and PKA signaling. As a result, transcriptional coactivator PGC1α was deacetylated and activated, leading to increases in the expression of genes linked to the complete oxidation of fatty acids. Overall, the authors concluded that oleic acid augmented rates of fatty acid oxidation in a SIRT1-PGC1α-dependent manner, explaining, at least in part, some of the protective effects of this fatty acid against inflammation, dyslipidemias, and insulin resistance, which may influence lipid homeostasis [167]. Such a profile marks oleic acid from SFAs, which is deprived of these potentially beneficial effects.

In addition, Thandapilly and colleagues demonstrated, in a model of rodent with diet-induced obesity, that oleic acid improved diastolic heart function. Oleic acid also showed the ability to reduce levels of inflammatory markers such as TNFα, suggesting that this may contribute to the observed oleic acid-mediated cardioprotection [168].

Indeed, proinflammatory cytokines, IL6 and TNFα, appeared markedly reduced in mice submitted to a sepsis treated for eight days with omega 9 (0.28 mg/100 μL). Conversely, anti-inflammatory cytokine IL10 was increased in the septic mice receiving omega 9. The authors suggested the involvement of the PPARγ pathway [169].

In summary, clinical and preclinical evidence suggests the necessity of further examination in order to clarify the complex effect of oleic acid on the cardiovascular system. The focus on the main operating conditions adopted for EVOO production and/or storage includes influence on the initial concentration of health compounds and on the kinetics of their degradation during storage. 

## 5. Focus on The Main Operating Conditions Adopted for EVOO Production and/or Storage: Influence on The Initial Concentration of Health Compounds and on The Kinetics of Their Degradation during Storage

According to Nicoli et al. 2012 [170], “shelf life” can be defined as a finite length of time after production (in some cases, after maturation or aging) and packaging during which the food product retains a required level of quality under well-defined storage conditions. 

With regards to EVOO, its shelf-life is directly linked to the occurrence of oxidation processes with a subsequent progressive degradation of the majority of both the saponifiable and the unsaponifiable fraction responsible for the healthy and nutraceutical properties attributed to EVOO. As reported in literature, EVOO shelf life has been assessed at 12–18 months [171], even if it has been shown that when it is properly stored in well-sealed packages, this product can reach the second year of storage, preserving the concentration of active health compounds and thus maintaining its nutraceutical and sensorial properties unaltered to the greatest possible extent [172].

However, the quality of EVOO in terms of both chemical compositions and sensorial expression depends on a process that begins with the olive ripening and finishes with the packaging. Thus, agronomical practices, raw materials, harvesting, fruit storage, and extraction technology, as well as oxygen, light, and temperature during storage, have to be considered in order to correctly estimate the nutraceutical, nutritional, and sensorial value [173,174,175].

**Figure 2:**
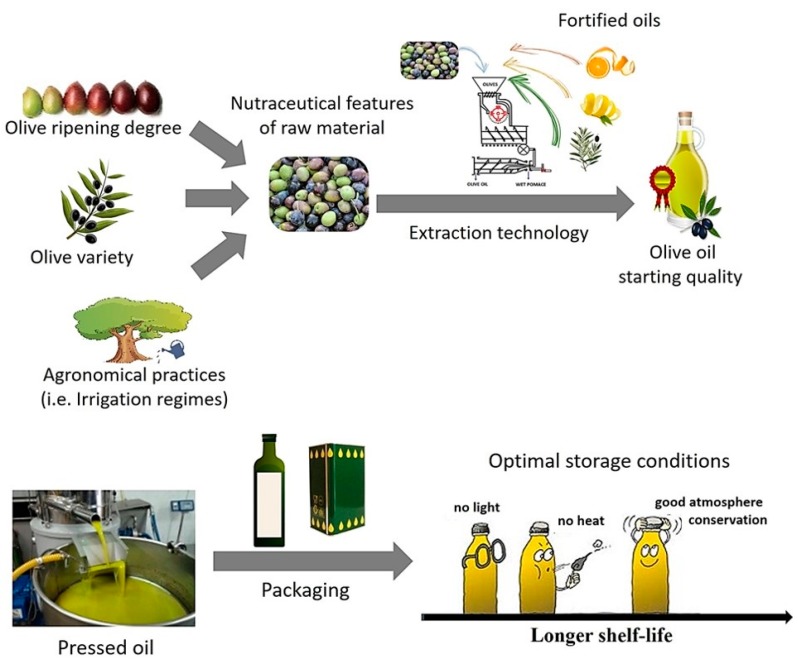
Main parameters that can influence olive oil shelf life: Characteristics of olive oil before storage and storage conditions.

Based on a critical analysis of recent scientific literature, Figure 2 illustrates the main factors that can directly influence the olive oil composition (i.e., saponifiable and unsaponifiable fractions) during production as well as the degradation rate of main health compounds during storage. 

### 5.1. Chemical Composition of Olive oil at Starting of Storage Time

The chemical and organoleptic quality of olive oil depends on several factors, such as the geographical location of the olive grove, the chemical and microbiological composition of the soil, the evolution of the climatic conditions during fruit ripening, and the extraction process [176,177,178].

Among the several variables that could potentially determine the quality of this product, the oil composition can be greatly affected not only by the cultivar (genetic variability) as well as the ripening degree but also by the cultivation techniques (i.e., irrigation system) and the climatic conditions occurring in a specific crop season.

#### 5.1.1. Characteristic of Raw Materials: Olive Cultivar, Ripening Degree, and Agronomic Practices

The oxidative stability of olive oil with respect to other vegetable oils is mainly due to its fatty acid composition, to the high MUFA/PUFA ratio in particular, and to the presence of minor compounds (i.e., polyphenols, carotenoids) that play a main role in preventing oxidation [173]. 

The expression of phenolic compounds in olive fruit is predominately driven by genetic factors, and large differences exist between olive cultivars [179]. In all cultivars, Ole and HT are the major phenolic compounds, but their concentrations vary considerably between cultivars at the same degree of ripeness [180]. 

During fruit ripening and processing, many chemical and enzymatic transformations that affect the accumulation of phenols inside the olives may take place [181]. In particular, due to the transformation of more structured compounds, phenols with a low molecular weight are produced [176]. As a consequence, the quality, sensory properties, oxidative stability, and the nutritional value of the olive oil can change considerably [177,178,182,183].

While the green or turning-color of olives creates a product characterized by bitter notes due to a higher presence of phenolic components (i.e., oleocanthal), the more acute and pungent notes are due to Tyrosol and its derivatives such as deacetoxy-ligstroside. Furthermore, some authors observed that the phenolic concentration of the olive fruit increases with ripening, reaching a maximum at the “half pigmentation” stage, after which it rapidly decreases [176]. This evolution could explain why some researchers report that the phenolic concentration increased with the ripening degree of the olives [184], while others observed an opposite evolution [176,185]. 

Finally, the environmental conditions (especially light) as well as the type of fertilization also deeply influences phenolic biosynthesis in plants [186]: While the yield of oil extracted from olive fruits belonging to the same cultivar and coming from the same orchard increased with the ripening degree of the milled fruits [180], according to Caruso and co-workers [187], the olives harvested on the same date from irrigated plants produced more oil than those coming from non-irrigated trees. Furthermore, agronomical practices seems to also influence the nutraceutical profile of extracted oil: Olives harvested from irrigated plants show a higher total phenol concentration value in the oil extracted than that obtained by milling fruits from non-irrigated trees [188], and the organic fruits have a higher phenolic content than conventional ones [186].

#### 5.1.2. Extraction Technology 

One of the most important industrial criticisms in the olive oil production is the low efficiency of current extraction techniques [189,190]. Nowadays, several studies have pointed out the importance of the different virgin olive oil processing stages on the extraction yield as well as the minor composition found in the final product, and the most used solution for improve extraction is increased malaxation time and/or temperature [191,192]. 

Scientific data report that milling and malaxation are the technological unit operations that most affect the quality of EVOO and the concentration of phenolic compounds and carotenoids, which are the main antioxidants of virgin olive oils [193,194,195,196]. During malaxation, the crushed olive paste is mixed slowly to promote coalescence, thus improving the separation efficiency of the subsequent centrifugation. The most critical point of this step is the possible oxidation of the polyphenolic compounds, leading to an oil with lower sensory and nutritional properties as well as a reduction in shelf-life [197,198].

Recently, Zinnai and co-workers set up an innovative system based on the direct addition of a cryogen (CO_2,s_) to olives during pre-milling phase, observing positive effects on the concentration of polyphenols and vitamin E [190,199]. 

Furthermore, in recent years, the development of new extraction methods based on the production of functional foods enriched with natural antioxidants has been demonstrated to be a promising potential application for the stabilization of olive oil and the increase of its shelf life [89,200]. 

It is worth mentioning that, due to their healthful and nutritional effects, considerable attention has been recently focused on identifying natural sources of antioxidants and improving their extraction processes—in particular olive oil by-products [200,201]—and fruit skin was also considered to produce enriched olive oils with an higher content of antioxidants compounds and, consequently, an improved nutraceutical profile [202]. 

### 5.2. Main Parameters Affecting the Degradation Rate of Health Compounds During EVOO Storage

Generally speaking, during storage the olive oil chemical composition (i.e., MUFA/PUFA ratio and concentration of minor compounds such as polyphenols and carotenoids) is influenced mainly by the final balance between oxidative degradation and antioxidant activity due to the presence of both tocopherols and phenolic compounds. In this context the lipid fraction shows the highest sensitivity to oxidative degradation with the subsequent development of off-flavors caused by the production of carbonyl and aldehyde compounds and the final occurrence of the typical “oxidative rancidity”. In addition, auto-oxidation based on a free radical mechanism starting from the formation of hydroperoxides induced by the initial oxygen availability further improve the degradation rate of the stored olive oil. 

While auto-oxidation can also be ruled out in the absence of light, this process appears to be accelerated by the action of natural photosensitizers such as chlorophyll, which reacts with triplet oxygen to form excited state singlet oxygen. In this context, the storage and packing conditions of olive oil become of primary importance [203].

#### 5.2.1. Influence of Storage Atmosphere 

Until now, many experimental studies have been carried out to verify the real effectiveness of the use of inert gases (i.e., nitrogen) in the head-space of the containers to improve the stability and the shelf life of the stored olive oil, thus slowing down its oxidative changes [193]. 

In a recent paper, Sanmartin and co-workers verified the possibility of using Ar and CO_2_ as head-space gases for the long-term storage of olive oil in order to slow down its oxidative degradation [174]. After 250 days of storage in the dark at a controlled temperature (12 ± 1 °C), the authors concluded that replacing air with Ar or CO_2_ in the headspace of the container during storage can significantly reduce the oil oxidation rate, thus preserving, as much as possible, the compositional, nutritional, and organoleptic qualities of the oil. In regard to chemical composition, while at the end of the observation period, the oil stored under CO_2_ appeared to be very similar to that stored in Ar atmosphere, it was significantly different with regard to sensorial characteristics. In particular, CO_2_ determined a negative organoleptic interference that would not support its use for the long-term storage of EVOO. Therefore, Ar treatment appears to be the best solution alternative to nitrogen to preserve the quality of the EVOO over time. 

#### 5.2.2. Characteristics of Packaging and Storage Temperature 

As discussed previously, among all the operating conditions that can influence the degradation rate of an olive oil, oxygen availability appears to be of primary importance, followed by the light exposure level. The presence of metal compounds must also be taken in account as they can play the role of activators of oxidative degradative reactions [173,175,204], thus reducing the concentration of active health compounds. 

It appears of primary importance, therefore, to carefully select the packaging materials with regard to the specific protection provided, together with the storage conditions to be adopted in order to preserve the nutraceutical features showed by the oil at the start of the storage time to the maximum extent. 

The main characteristics of the most widely used packaging materials for the storage of olive oil, together with a description of their specific functionality in terms of olive oil preservation, are given in Table 5. 

In particular, metal containers can provide total protection against light, oxygen, and water vapor. In order to avoid the activation of oxidation by metallic catalysis, it is possible to opt for tin plate or tin-free steel based on chromium instead of aluminum or aluminum alloys. In addition, while the inside of the tin can be coated with resins to protect the metal surface against corrosion, particular attention should be paid, in this case, to the main concern related to the leaching of unsafe chemical compounds from food contact materials (FCM) into the stored oil. Glass represents a good barrier against moisture and gases without leaching [201], but transparent bottles cannot protect the olive oil from photo-oxidation [203]. For this reason, glass containing specific additives to significantly reduce the transmittance of light in the UV range have been created [205].

To determine the effects of packaging on the commercial life of olive oil, several studies have been carried out, and different containers such as clear and dark bottles, polyethylene, and tin containers have been taken into consideration [203,206], and the storage stability of oils in tin or stainless containers and in dark glass was the highest [203]. 

Besides the type of packaging, storage temperature can also influence the degradation rate of stored olive oil [173,206], obtaining a longer shelf life when a lower temperature was adopted during storage.

In a recent paper, Sanmartin and co-workers [173] evaluated the effects of packaging and storage conditions on an EVOO as it occurs in most points of sale: the storage of oil in tanks under nitrogen for a more or less long time (also for several months), after which the oil is packaged and sold. Interestingly, st the end of the observation period, the authors observed that the storage conditions can not only prevent oxidation processes from occurring but they can even be usefully implemented to slow down or almost block these processes in the case of oil in which the oxidative processes had already started.

## 6. Conclusions

In accordance with clinical and preclinical evidence, regulatory agencies recognize the potential interesting and beneficial effects of EVOO on the cardiovascular system, particularly those aimed at the reduction of risk factors in which oxidative stress and inflammatory processes play a critical role. Despite a clear vision for this functional food, there seems to be a nebulous view on the main constituents, polyphenols, vitamin E, and finally oleic acid. Indeed, an analysis of the clinical and preclinical studies shows the necessity for further examination in order to fully understand their contribution to the overall nutraceutical and nutritional value of EVOO. Moreover, several operating conditions, from production up to storage, can deeply influence the shelf life of olive oil in terms of both chemical composition mainly related to health compounds (i.e., MUFA/PUFA ratio; concentration of minor compounds such as polyphenols and carotenoids) and sensory quality, therefore, these aspects need to be carefully considered. Indeed, great efforts are being made in the agronomic field to optimize these conditions.

## Figures and Tables

**Table 1:** Main and secondary keywords used for the literature search.

Main Key Words	Secondary ^1^ Key Words
EVOO^2^ production EVOO storage Fortified oils EVOO	Olive ripening Olive agronomical practices Packaging Storage conditions
Hydroxytyrosol Tyrosol Oleuropein Olive oil polyphenols Oleic acid MUFA^3^ Olive oil Vitamin E Tocopherols Tocotrienols	Nutraceutical properties Antioxidant Anti-inflammatory Cardiovascular effects Metabolism Bioavailability Clinical trials Preclinical studies

^1^ Secondary key words were utilized in combination with the main key words listed in left column. ^2^ Extra Virgin Olive Oil ^3^ Mono Unsaturated Fatty Acids.

**Table 2:** List of clinical trials with Olive Oil Polyphenols.

Health Status	N.^1^	Study	Treatment	Efficacy	Ref.
Hypercolesterolemia	4	Randomized, double-blind, placebo and active comparator (Armolipid Plus) controlled study	Food supplement called Body Lipid, containing monacolin K (10 mg), berberine (500 mg), coenzyme Q10 (2 mg) and HT (5 mg)	+	[60]
		Randomized, controlled, double-blind, crossover human trial	VOO containing polyphenols 80 mg/kg, or 500 mg/kg, or a mixture from VOO and thyme (500 mg/kg, 1:1)	+	[61]
		Randomized, double-blind crossover, controlled trial	olive oils with different phenolic contents, 80 or 400 ppm	+	[62]
		Observational non-randomized study	Cholesfytol (10 mg Monacolin K and 5 mg HT)	+	[63]
Obesity	1	Randomized, double-blinded, placebo-controlled, crossover	51.1 mg oleuropein, 9.7 mg hydroxytyrosol	+/−	[64]
Metabolic syndrome	2	Randomized double-blind placebo-controlled trial	Cholesfytolplus capsule (10.82 mg Monacolins and 9.32 mg HT)	+	[65]
		Randomized double blind placebo controlled randomized trial	Cholesfytolplus capsule (10.82 mg Monacolins and 9.32 mg HT)	+	[66]
Hypertension	2	Randomized, double-blind, controlled, crossover trial	Phenolic-rich olive leaf extract (136.2 mg Ole and 6.4 mg HT per day)	+	[47]
		Randomized, double blind, crossover trial	Virgin OO enriched with polyphenols-961 mg/kg	+	[45]
Arterial stiffness	1	Randomized double-blind placebo-controlled trial	Standardized olive fruit extract 250 mg (50 mg HT) or 500 mg (100 mg HT)	+	[67]
Healthy volunteers	9	Randomized double-blinded, placebo-controlled crossover trial	15 mg/day of HT	+	[68]
		Randomized, cross-over, placebo-controlled and double-blind trial group.	25 mg/day HT (extract of olive mill wastewater called Hytolive)	+	[69]
		Randomized, double-blind, placebo-controlled, cross-over trial	51 mg Ole and 10 mg HT	+	[70]
		Randomized double-blind, placebo-controlled study	5 and 25 mg/d HT	−	[44]
		Randomized double-blind placebo-controlled study	Virgin OO enriched with polyphenols—5358 mg/L	+	[71]
		Randomized, double-blind crossover, controlled trial	OO with a low polyphenol content (2.7 mg/kg) or a high phenolic content (366 mg/kg)	+	[72]
		Randomized, double-blind crossover, controlled trial	OO with low (2.7 mg/kg of olive oil), medium (164 mg/kg), or high (366 mg/kg) phenolic content	+	[73]
		Randomized, double-blind crossover, controlled trial	OO with low (2.7 mg/kg), medium (164 mg/kg), or high (366 mg/kg) phenolic content	+	[74]
		Randomized, double-blind crossover, controlled trial	OO with low (0 mg/kg), medium (68 mg/kg) or high (150 mg/kg) phenolic content	+	[75]

Abbreviations: + = cardioprotective effect(s); +/− = partial cardioprotective effect(s); − = loss of cardioprotective effect(s). **^1^** Number of clinical trials examined

**Table 3:** List of clinical trials with Vitamin E.

Health Status	N.^1^	Study	Treatment	Efficacy	Ref.
Healthy subjects	9	Prospective cohort study	Vitamin E (as α-tocopherol equivalents)	+	[106]
		Prospective cohort study	Vitamin E	+	[107,108]
		Prospective cohort study	Vitamin E	+	[110]
		Follow-up	Vitamin E	+	[7]
		Cohort study	Vitamin E supplementation with food intake	+	[112]
		Cohort study	Vitamin E	−	[120]
		Randomized, double-blind, placebo-controlled, cross-over trial	Vitamin E alone, vitamin E + other antioxidants	+	[125]
		Randomized, double-blind, placebo-controlled primary prevention trial	Vitamin E	−	[118]
Healthy subjects (platelet aggregation induction)	2	Randomized, double-blind, placebo-controlled, cross-over trial	α-, γ-, δ-tocopherol	+	[114,115]
High cardiovascular risk	1	multicenter, parallel group, randomized controlled clinical trial	Vitamin E	−	[124]
Patients with evidence of vascular disease or diabetes	2	Randomized, double-blind, placebo-controlled, cross-over trial	Vitamin E	−	[122,123]
Coronary atherosclerosis	1	Double-blind, placebo-controlled study with stratified randomization	Vitamin E	+	[111]
Patients surviving after recent myocardial infarction (3 months)	1	Multicenter, open-label design, in which patients were randomly allocated	Vitamin E	−	[121]
Postmenopausal women	1	Prospective cohort study Follow-up	Vitamin E	+	[109]
Hemodialysis patients with pre-existing cardiovascular disease	1	Randomized, double-blind, placebo-controlled, cross-over trial	Vitamin E	+	[113]
Type 2 diabetes	1	Randomized, double-blind, placebo-controlled, cross-over trial	Tocotrienols + tocopherols	+	[117]
Metabolic syndrome	1	Randomized, double-blind, placebo-controlled, cross-over trial	γ-tocopherol, α-tocopherol	+	[116]

**Abbreviations:** + = cardioprotective effect(s); − = loss of cardioprotective effect(s). **^1^** Number of clinical trials examined.

**Table 4:** List of clinical trials with oleic acid.*.

Health Status	N.^1^	Study	Treatment	Efficacy	Ref.
CVD risk subjects	1		32 g/day of EVOO	+	[164]
Hypercholesterolemic patients	1	Randomized crossover study	Experimental diet enriched with oleic acid	+	[162]
Patients with left ventricular hypertrophy risk	1	Longitudinal cohort		-	[163]
Healthy subjects	5	Randomized control trial	Milk enriched with oleic acid and/or PUFA	+	[160]
		Control non-randomized	Milk enriched with oleic acid and/or PUFA	+/−	[160]
Hypercholesterolemic patients	1	Randomized control study	Milk enriched with oleic acid and/or PUFA	+	[160]
Metabolic syndrome subjects	1	Randomized control study	Milk enriched with oleic acid and/or PUFA	+	[160]
Peripheral vascular disease patients	1	Randomized control study	Milk enriched with oleic acid and/or PUFA	+	[160]
Myocardial infarction patients	1	Randomized control study	Milk enriched with oleic acid and/or PUFA	+	[160]

**Abbreviations:** + = cardioprotective effect(s); +/− = partial cardioprotective effect(s); − = loss of cardioprotective effect(s). **^1^** Number of clinical trials examined

**Table 5:** Packaging materials most used for olive oil storage and their characteristics.

Packaging Material	Barrier Against Gases	Light Protection	Absence of Metals	Interaction FCM/oil
Glass	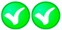	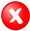	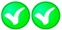	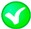
Glass + additives anti-UV	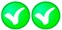	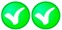	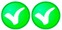	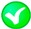
Aluminium/Aluminium alloys tin-plate	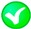	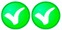	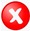	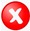
Chromium tin-free steel	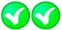	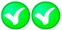	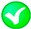	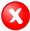
Tin-plate + resins coating	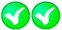	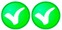	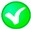	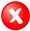
Polyethylene	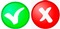	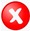	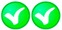	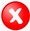
